# Mapping landscape changes to address dengue fever risk in Laos and Thailand

**DOI:** 10.1016/j.crpvbd.2026.100351

**Published:** 2026-01-13

**Authors:** Muhammad Umar, Sobia Asghar, Sumaira Zafar

**Affiliations:** a Institute of Nutrition, Mahidol University, 999 Phutthamonthon 4 Road, Salaya, Phutthamonthon, Nakhon Pathom, 73170, Thailand; b Department of Agricultural Sciences and Technology, Atta-ur-Rahman School of Applied Biosciences, National University of Sciences and Technology, Islamabad, Pakistan

**Keywords:** Land use and land cover changes, LULC, Dengue vector habitats, Forests, Rubber plantations, *Aedesaegypti*, *Aedes albopictus*, Urbanization

## Abstract

Land use and land cover (LULC) are changing rapidly worldwide, with climate change making living conditions uncomfortable for humans while creating favorable environments for dengue vectors, *Aedes aegypti* and *Aedes albopictus*. Laos and Thailand are endemic to dengue, with frequent outbreaks reported. Cleared forests allow vectors to interact with nearby human hosts, potentially transferring viruses from zoonotic reservoirs. Urban areas are often built at the expense of natural hydrology, forests, or farmland. These areas, known as urban heat islands, absorb heat because of construction materials that retain warmth. This, along with water containers or paddles, creates ideal breeding grounds for dengue vectors. Additionally, rubber plantations have been identified as breeding sites for vectors, which can worsen endemic conditions. This study aimed to map LULC changes associated with dengue vector proliferation from 2001 to 2025 using Landsat satellite images (1 to 8). Random Forests classification, a supervised machine learning method, was used to delineate LULC patterns. Satellite-derived LULC data showed a significant decrease in forest cover, an increase in settlements, and a shift in agricultural crops from food crops to cash crops, specifically rubber, confirming changes previously linked to dengue vector ecology. These findings raise serious concerns for public health agencies in both countries and provide valuable insights for cross-sectoral policies in public health, urban planning, forestry, and agriculture sectors.

## Highlights

• Random Forest classifier achieved 80–84 % accuracy in classifying LULC changes in Laos and Thailand during 2001–2025.
• Forest cover declined in all four provinces studied, with increases in settlements and cash crop (rubber) plantations.
• Shift to monoculture plantations may reduce biodiversity and create new mosquito breeding habitats.
• Built-up areas expanded around provincial capitals, particularly in Savannakhet (Laos) and Ubon Ratchathani (Thailand).
• Urbanization may increase artificial breeding sites and may influence mosquito biting and virus replication.

## Graphical abstract

**Figure:**
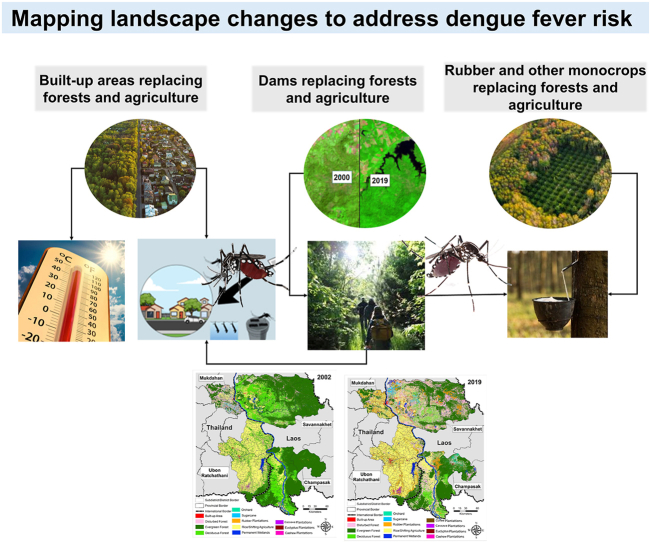

## 1 Introduction

Dengue is prevalent in Southeast Asia, with an estimated 21.1 million infections reported annually (Brady et al., 2012; Stanaway et al., 2016; Zeng et al., 2021). In Laos, dengue outbreaks recur every few years, placing over 3.9 million people across nine out of 17 provinces at risk (Mayxay et al., 2013). In Thailand, where dengue is hyperendemic with all four serotypes cocirculating, approximately 20,000–140,000 cases are reported annually, and the entire population remains (Lim et al., 2025; Nonyong et al., 2021; Thisyakorn et al., 2022). Human-driven changes in both countries modified the spatial heterogeneity of land use and land cover (LULC), potentially increasing risk based on vector distribution and abundance because land cover determines suitable habitats for dengue vectors, whereas land use affects host distribution and influences host-vector interactions, including biting rates (Hashizume et al., 2012).

Dengue is mainly transmitted by *Aedes aegypti* (Gubler, 2011) and *Aedes albopictus* (Kraemer et al., 2015), mosquito species whose breeding sites are closely linked to human-altered environments. *Aedes aegypti* is highly anthropophilic, biting multiple times during the day and predominantly feeding on humans (WHO, 2009), which amplifies transmission in urban areas. By contrast, *Ae. albopictus* is more opportunistic, feeding on both humans and animals (Pereira-Dos-Santos et al., 2020), which enables persistence in rural, vegetated landscapes and facilitates transmission at the forest-village interface. Human settlements are a habitat use type that supports *Ae*. *aegypti* proliferation (Ooi and Gubler, 2008; Wan-Norafikah et al., 2012). These mosquitoes lay drought-resistant eggs on water-holding containers (natural and artificial), near water surfaces or on dry surfaces, estuaries, tree holes, leaf axils, and soils, and can hatch under favorable conditions such as rain (Sallam et al., 2017). Such ecological flexibility allows both species to rapidly exploit habitats generated by LULC changes, whether through artificial containers in urban settlements or tree holes and crop-associated water bodies in rural plantations. Converting land covers into different land uses modifies the contact between susceptible hosts and infectious vectors (Vanwambeke et al., 2011), impacting disease distribution (Norris, 2004; Vanwambeke et al., 2006, 2007), such as forest conversion into rubber plantations, which has been reported from Laos and Thailand (Tangena et al., 2017b; Zafar et al., 2025). Several studies conducted in both countries provide evidence for the presence of these vectors in different LULC types; *Ae*. *albopictus* has been found in rubber plantations and secondary forests (Tangena et al., 2017a; Thammapalo, 2005; Zafar et al., 2025), while *Ae. aegypti* is found in urban areas, wetlands, and green spaces.

Mapping LULC changes is important not only for planning and conservation purposes, but also for monitoring shifts related to diseases like dengue, which depend on natural and man-made conditions. This study explores how LULC transformations might influence the ecological context of dengue transmission in Laos and Thailand, two countries facing high disease burdens over the years. Rather than proving direct causality, we hypothesize that specific LULC changes can create environmental conditions that affect vector habitats and, in turn, dengue risk. Laos is among the least studied countries in the region for dengue fever risk and also for LULC changes. This research builds on earlier work that collected dengue mosquito vectors and their larvae from various LULC types and reported dengue fever cases close to different LULCs. It is crucial since this may help understand how LULC changes influence dengue vector habitat distribution. By documenting these changes, the study underscores the broader impacts of human activities on the environment and the conditions favorable for dengue vectors. The novel aspect of this research is analyzing LULC changes over time alongside shifts in dengue epidemiology, offering valuable insights into human impacts on landscapes. These findings can serve as a valuable reference for policymakers, urban planners, and conservationists to develop strategies that balance development with ecological sustainability.

We focused on dengue vectors, specifically *Ae. aegypti* and *Ae. albopictus*, the main species responsible for dengue transmission in Laos and Thailand. Dengue is endemic in Laos and hyperendemic in Thailand, with major outbreaks occurring regularly, making it a significant public health issue. Both *Aedes* species are highly connected to human-modified environments; *Ae. aegypti* thrives in urban and peri-urban settings, while *Ae. albopictus* is associated with rural, vegetated areas, including rubber (Thammapalo, 2005) and durian orchards (Ratisupakorn et al., 2021). These ecological and behavioral differences highlight why land conversion, such as deforestation, agricultural expansion, and urbanization, can shift vector population dynamics differently across regions. In Thailand, urban cycles are largely driven by *Ae. aegypti*, whereas in Laos, peri-urban and rural landscapes provide favorable habitats for *Ae. albopictus*, underscoring the need for context-specific analyses. By concentrating on dengue, we aim to interpret associations between LULC changes and disease risk with respect to known dengue vector habitat preferences and transmission cycles.

## 2 Materials and methods

### 2.1 Study area

**Fig. 1:**
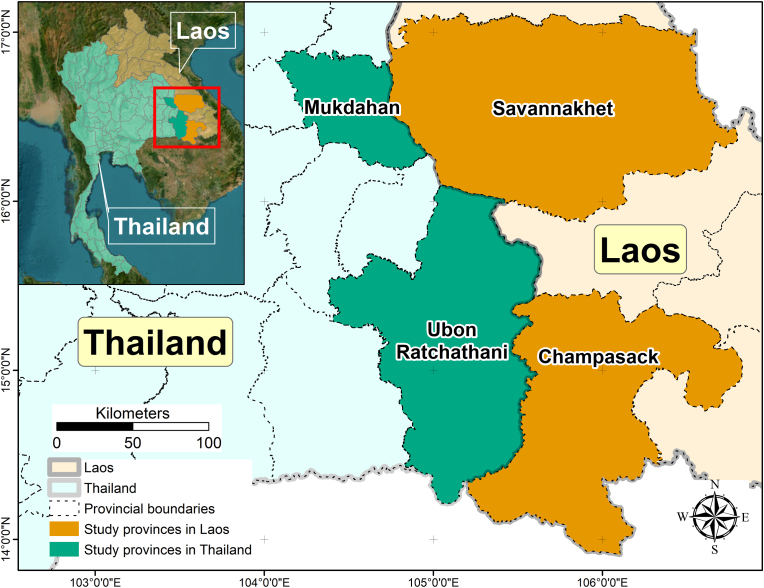
Map showing the study provinces in central and southern Laos and in northeastern Thailand.

This study was conducted in four neighboring provinces along the Mekong River in Laos and Thailand, Savannakhet, and Champasak in Laos, and Mukdahan and Ubon Ratchathani in Thailand (Fig. 1). These provinces are quite similar in terms of culture, language, and history; however, they differ in socio-economic and political conditions, and are expected to vary in climate vulnerability, adaptive capacity, geographical and ecological diversity, and socio-economic status. The region has a tropical climate with a dry cool season from mid-October to mid-February, a dry hot season from mid-February to mid-May, and a monsoon rainy season from mid-May to mid-October with higher rainfall, humidity, and temperatures.

### 2.2 Data collection

In this study, images from the Landsat Earth Observation Satellite Series, Landsat 7-Enhanced Thematic Mapper Plus (ETM+) for 2001–2003, Landsat5Thematic Mapper (TM) for 2004–2011, and Landsat8-Optical Land Imager and Thermal Infrared (OLI-TIRS) for 2013–2025, were used to map temporal changes in land use and land cover (LULC) of all four provinces (USGS, 2021). In Thailand and Laos, where cloud cover is common during the monsoon season (June to November), Sentinel-1 imagery from that period was used to map wetlands.

### 2.3 Delineation of land use and land cover (LULC)

The LULC of the provinces has undergone significant transformation over the past few decades. Changes were identified and mapped for the years 2001–2025. Landsat TM, ETM+ and OLI-TIRS images were classified using a supervised machine learning method called Random Forests classification. Two image processing tools, R and SNAP, were employed.

**Fig. 2:**
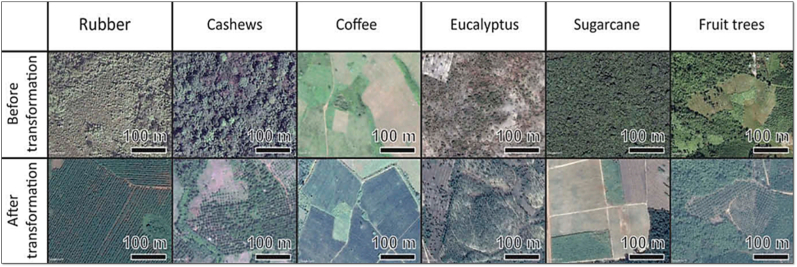
Identification of LULCs from Google Earth.

**Fig. 3:**
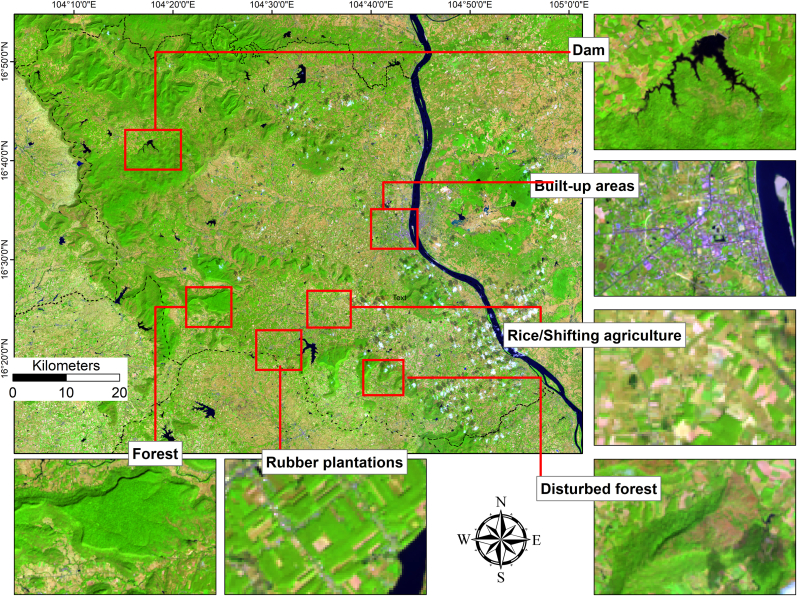
Identification of LULCs from false color composite of Landsat satellite images.

To ensure comparability across different sensors and time periods, all Landsat images (TM, ETM+, OLI-TIRS) were atmospherically corrected and converted to surface reflectance prior to classification. Only images from the dry season (December-February) were used where possible to minimize seasonal effects and avoid cloud cover. Band combinations with consistent spectral information across sensors (e.g. red, green, blue, near-infrared, shortwave-infrared) were selected to reduce differences caused by varying sensor specifications. The classification of satellite images required ground control points (GCPs) as training areas for LULC. GCPs were collected from multiple sources, including ground surveys, high-resolution Google Earth imagery, Google Street View images (Fig. 2), visual interpretation of Landsat images (Fig. 3), and previous studies with published GCPs (Hurni and Fox, 2018). There are reports indicating that accurate supervised classification depends on sufficient GCPs that reflect the class’s spatial and temporal diversity, as well as the spectral temporal similarity of classes (Foody and Arora, 1997; Hurni et al., 2017; Heydari and Mountrakis, 2018). For classes with similar spectral and temporal characteristics, such as rubber, forests, crops (cassava, coffee, rice, sugarcane), and built-up areas (often mixed with wet soils and shadows), enough GCPs were collected. The selected LULC classes mapped (Table 1) for each province identified from Google Earth and false color composite of satellite image are shown in Fig. 2, Fig. 3. The LULC classes in Fig. 2 show the change in LULC between 2001 and 2025, with most of them replacing forest and agricultural areas, specifically rice.

**Table 1:** Land use and land cover mapped in each study province of Laos and Thailand.

Land use and land cover mapped	Thailand		Laos	
	Ubon Ratchathani	Mukdahan	Champasak	Savannakhet
Built-up area	✓	✓	✓	✓
Rubber plantations	✓	✓	✓	✓
Rice/shifting agriculture	✓	✓	✓	✓
Eucalyptus plantations	✓		✓	
Evergreen forest	✓	✓	✓	✓
Disturbed forest	✓	✓	✓	✓
Deciduous forest	✓	✓	✓	✓
Sugarcane crop	✓	✓	✓	✓
Permanent wetland	✓	✓	✓	✓
Cassava plantations	✓			
Cashew plantations			✓	
Coffee plantations			✓	
Orchard plantations			✓	

### 2.4 Random Forests classification

The supervised machine learning algorithm Random Forests (RF) was used, as it has been shown to achieve higher classification accuracy in ecological studies (Cutler et al., 2007; Schneider, 2012). RF builds multiple decision trees that learn from data and combine their results to improve accuracy. Compared to traditional methods like maximum likelihood classification, RF handles noise better, manages non-linear relationships in the data, and makes no assumptions about data distribution (Cutler et al., 2007). RF is well-suited to large datasets because of its flexible parameters and computational efficiency (Rodriguez-Galiano et al., 2012; HUB Geomatics Lab, 2015). RF randomly selects subsets of satellite bands as features at each decision node, with a fixed number of features. In this study, 500 decision trees were used after testing whether increasing the number of trees from 500 to 1000 and 10,000 improved accuracy; results showed no significant gains (HUB Geomatics Lab, 2015). Water bodies were mapped using Landsat and Sentinel-1 images. The Normalized Difference Water Index (NDWI) was used to extract water extent from optical images ($NDWI=\frac{Green-NIR}{Green+NIR}$) with green and near-infrared bands (NIR) from Landsat. NDWI values range from −1 to +1, with values ≥ 0.5 indicating water.

SAR images from Sentinel-1 during the dry season (December-February) were analyzed for water detection. Only the “VV” polarization signal was used, representing vertically transmitted and received signals. Care was taken to consistently transmit/receive polarizations across all images. Speckle noise in SAR images was reduced using a focal median filter, which computes the median value of each pixel and its neighbors.

The primary goal was to identify flooded areas; a simple threshold of −16 backscatter was applied to classify pixels as water, with darker areas indicating (Kuntla and Manjusree, 2020). This threshold was determined heuristically.

## 3 Results

The Random Forest classifier performed consistently well across all study sites and years. The overall accuracy for classifying satellite images from Mukdahan, Ubon Ratchathani, Savannakhet, and Champasak remained approximately 80–84% in all years (confusion matrices for all years are provided in Supplementary file 1). Overall classification accuracy of more than 80%, indicating a reliable separation of the LULC classes (see Supplementary file 1). Classification performance was generally stable over time, suggesting that the selected spectral bands and training samples were effective in capturing the characteristic signatures of each LULC type.

**Fig. 4:**
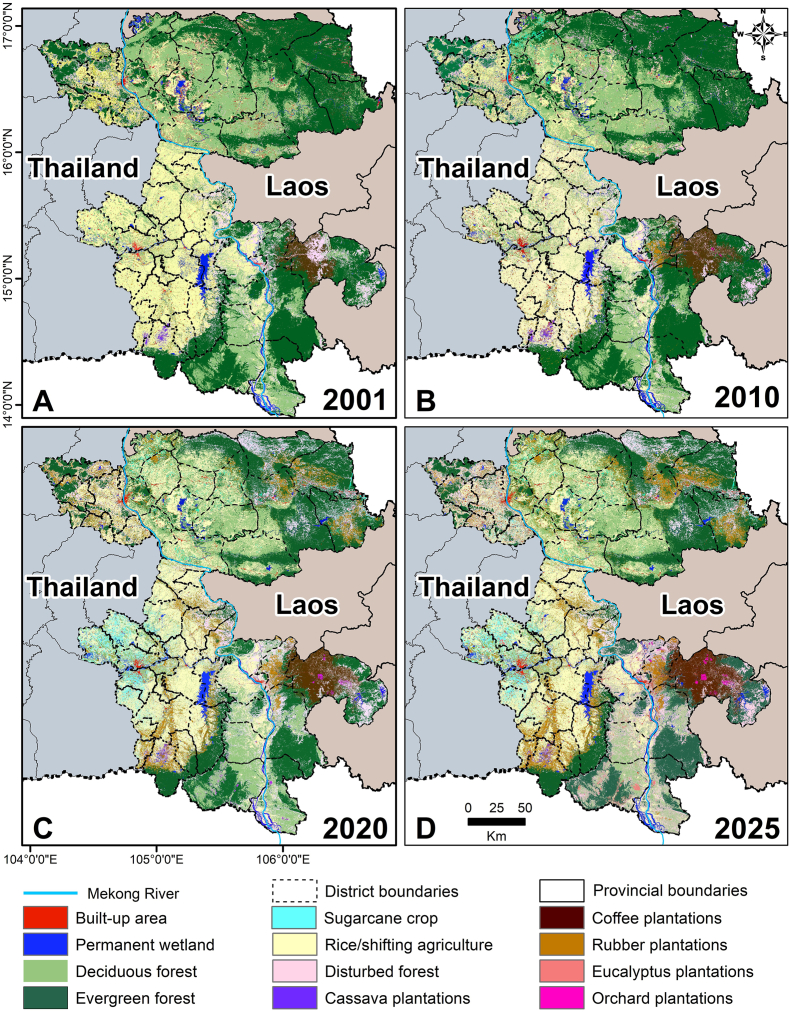
Land use and land cover (LULC) in Mukdahan and Ubon Ratchathani (Thailand) and Champasak and Savannakhet (Laos) in 2001 (**A**), 2010 (**B**), 2020 (**C**), and 2025 (**D**).

**Fig. 5:**
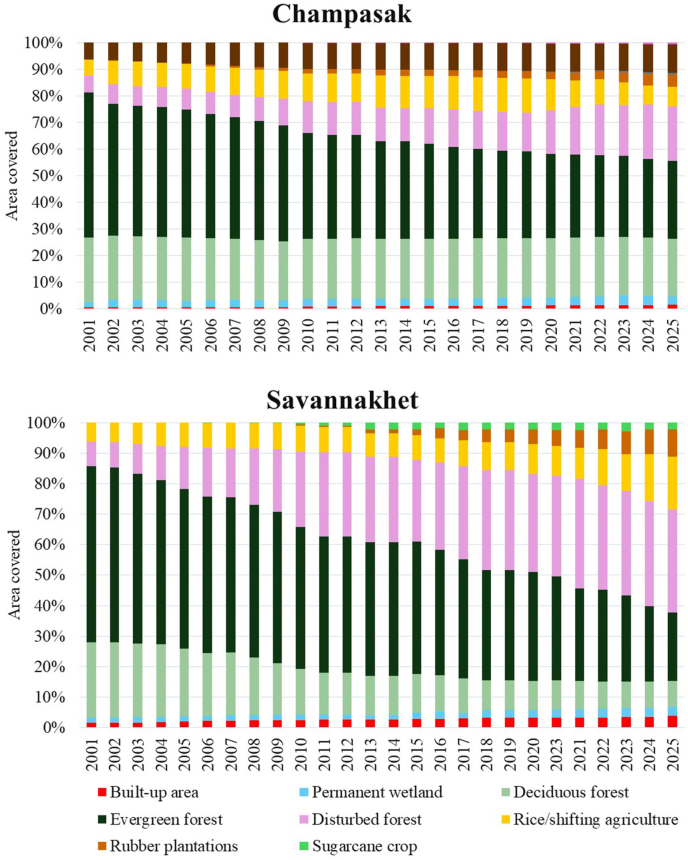
Land use and land cover (LULC) changes from 2001 to 2025 in Champasak and Savannakhet (Laos).

**Fig. 6:**
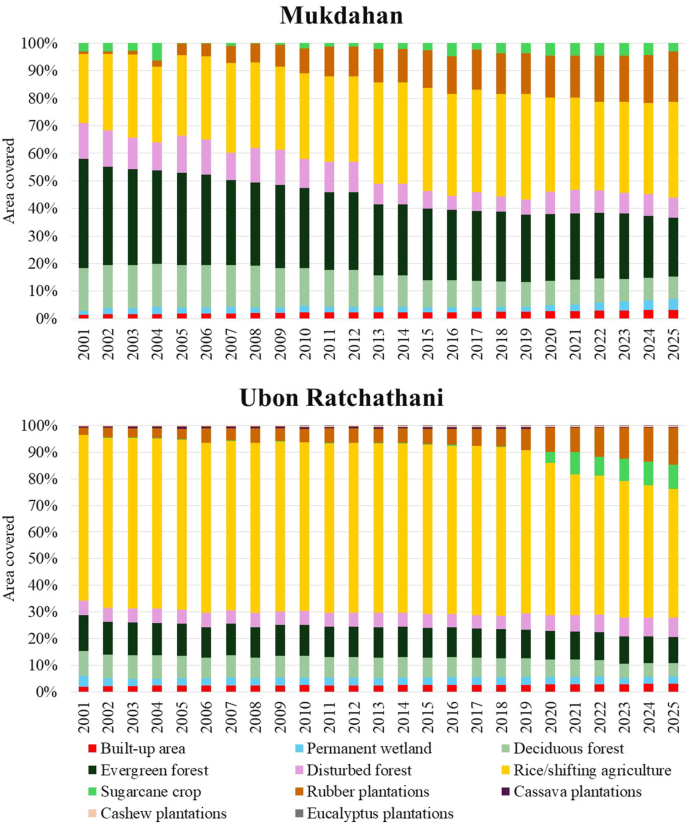
Land use and land cover (LULC) changes from 2001 to 2025 in Mukdahan and Ubon Ratchathani (Thailand).

The significant change in LULC brings other environmental effects, such as localized or regional weather changes, altered hydrology and water availability, impacts on the life cycle of the vectors and host distribution. The major LULC shifts in Laos and Thailand occurred between 2002 and 2014, followed by gradual transitions in recent years (Fig. 4, Fig. 5, Fig. 6). Spatial patterns of LULC change (2001 to 2025) are depicted in Fig. 4. In 2001, large areas of Savannakhet and Champasak (Laos) and Mukdahan and Ubon Ratchathani and (Thailand) were covered by deciduous and evergreen forests. By 2025, these forested areas had been significantly reduced, especially in Mukdahan and Savannakhet, replaced by agricultural land, rubber plantations, and other crops. Rubber plantations are clearly visible in northeastern Thailand and parts of southern Laos, while sugarcane, cassava, and rice dominate the plains. Urban and built-up areas expanded around provincial capitals, particularly in Savannakhet and Ubon Ratchathani. The trend in changes shown in the stacked bar charts (Fig. 5, Fig. 6), indicates forest cover declines are accompanied by increases in plantations and cropland.

### 3.1 Laos

In both provinces (Fig. 5), Champasak and Savannakhet, forest cover has steadily decreased during 2001–2025 and converted to disturbed forests, i.e. areas where natural forest structure and function have been altered by human activity or natural events. This conversion is directly attributable to mechanisms such as mining activities (Schneider, 2012), dam construction, and forest thinning (Morris and Fiorino, 2008).

In Savannakhet Province, deciduous and evergreen forests cover declined (from 24.9% in 2001 to 8.5% in 2025 and from 57.8% to 22.4%, respectively), with much of this loss occurring during 2008–2015, and disturbed forests cover increased (from 8.0% to 33.9%) (Fig. 5; see Supplementary file 2 for details). Champasak Province also experienced a decline in total forest cover (deciduous forests: from 24.3% to 21.8%; evergreen forests: from 54.5% to 29.3%). This province also experienced a 3-fold increase in disturbed forest (20.4%) in 2025 compared to the disturbed forest area in 2001 (6.2%), due to dam construction and other activities (Morris and Fiorino, 2008). In both provinces, most deciduous and evergreen forests were converted into disturbed forests, and later transformed into croplands (rice and sugarcane) and plantations (rubber, eucalyptus, coffee, cashew, and orchards). Rubber plantations continued to grow steadily, increasing to 9.0% of Savannakhet Province area and from 0.02% to 4.5% of Champasak Province area. Sugarcane cultivation expanded only in the northwestern districts of Savannakhet Province, showing a rise (from 0.07% to 2.2%) from 2008 to 2025. Urban areas also expanded but at different rates; there was an increase from 1.5% to 3.8% in Savannakhet and from 0.4% to 1.4% in Champasak. Smaller plantation types such as cashew, coffee, and eucalyptus contributed little to overall land cover but still replaced forests or shifting agriculture (see Supplementary file 2 for details).

### 3.2 Thailand

Thailand’s LULC transitions were mainly driven by the expansion of rubber plantations and the transformation of rice cultivation areas into cassava plantations, which were later replaced by rubber plantations (Fig. 6; see Supplementary file 2 for details). In Mukdahan Province, total forest cover sharply declined from 55.3% in 2001 to 29.5% in 2025, with significant losses in both deciduous (from 15.6% to 8.0%) and evergreen (from 39.78% to 21.5%) forests. Ubon Ratchathani Province also experienced reductions, with total forest cover decreasing from 22.8% to 15.0%. During 2001–2025, rubber plantations expanded to cover 18.3% of Mukdahan and 13.8% of Ubon Ratchathani, while rice in both provinces and cassava in Ubon Ratchathani alternated as the primary land uses. The stacked bars in Fig. 6 illustrate these changes; Ubon Ratchathani shifted from deciduous and evergreen forests to rice, then to cassava plantations, finally to rubber plantations, with cassava plantations reaching a peak around 2010 before sightly gradually decreasing. Urban expansion was modest but steady, increasing from 1.4% to 3.2% in Mukdahan and from 2.0% to 3.0% in Ubon Ratchathani. Other plantations, such as cashew and eucalyptus, remained in a small portion but contributed to the ongoing replacement of forests.

Collectively, these LULC changes demonstrate a significant shift from forested landscapes to agricultural, plantation, and urban areas and wetlands (dam construction), altering the ecological environments in which vector habitats are likely to develop.

## 4 Discussion

This study did not aim to establish a direct causality between LULC and dengue incidence. Instead, it identified and characterized LULC changes that influence the ecological context for dengue vectors and their virus transmission. Previous research, including our own epidemiological studies using the same dataset, has shown statistical links between LULC transformations and dengue cases in Laos and Thailand. Specifically, our work found that increases in built-up areas significantly raised dengue risk in Champasak and Savannakhet provinces of Laos by 6.8% and 4.9% per 1 km^2^, respectively. Similarly, in Thai provinces, the risk increased by 5.4% in Ubon Ratchathani and 1.8% in Mukdahan. The effects of agricultural LULC were more heterogeneous; for instance, rubber plantation expansion increased risk by 0.9% in Ubon Ratchathani but decreased it by 2.2% in Mukdahan, while sugarcane showed a strong positive association in Mukdahan (11.3% rise in risk). Conversely, increases in deciduous forest and temporary wetlands were generally related to decreased dengue risk (Zafar et al., 2025).

The present analysis is complementary, providing a spatially detailed view of LULC dynamics to support risk assessment and cross-sectoral policy decisions. Our study highlights how LULC changes reshape ecological systems, especially through forest loss and the expansion of rubber plantations, rice fields, and built-up areas. These shifts alter habitat availability and connectivity, which in turn affect the spatial distribution of ecosystems and habitats important to dengue vectors. These results align with broader scientific research on the ecological and epidemiological effects of such environmental modifications. In both Laos and Thailand, significant deforestation has been observed mainly due to agricultural expansion and plantation development. Such deforestation modifies microclimates and creates temporary breeding sites, like puddles and old tires, thereby increasing human exposure to vectors at forest edges, particularly *Ae. albopictus.* Prior studies from Laos (Tangena et al., 2016), and Malaysia (Brant, 2011; Brown et al., 2018), have documented that secondary forests, characterized by dense undergrowth, serve as primary habitats for *Ae. albopictus.* These environmental changes can disrupt natural ecosystems and alter mosquito habitats, potentially influencing disease transmission pathways. In Savannakhet Province, deforestation mainly results from disturbance rather than extensive forest clearing. Ongoing gold mining involves the removal of deciduous forest trees, creating large, circular cleared areas that have gradually undergone reforestation. This aligns with field studies in Laos showing that secondary forests provide habitats for *Ae. albopictus* (Tangena et al., 2017a).

The conversion of forests and other natural habitats into rubber plantations is a notable trend, as farmers are transitioning from diverse agricultural practices to monocultures such as eucalyptus, rubber, cashew, banana, and other orchards to secure economic stability. This process includes the reduction of biodiversity through monoculture cultivation, the use of pesticides that may disrupt natural mosquito control mechanisms, the creation of new breeding sites, such as latex cups for *Aedes* spp., and increased human activity in agricultural zones. A recent study mapping dengue risk in Laos and Thailand identified rubber plantations as being positively linked to dengue occurrence (Zafar et al., 2025). Similar associations have been documented in Phuket, Thailand (Thammapalo, 2005) and in other studies (Gratz, 2004; Vanwambeke et al., 2007), which highlighted the role of rubber plantations in creating suitable breeding habitats for *Aedes* spp. mosquitoes in latex cups filled with rainwater.

The expansion of rice cultivation and shifting agriculture in both countries is also linked to the construction of hydropower dams in Laos provinces, specifically, one in Champasak and four in Savannakhet. Additionally, the conversion of rice fields into cassava plantations and their subsequent transformation into rubber plantations reflect the economic pressures driving land use and land cover (LULC) changes. This pattern was especially evident in the Thai province of Ubon Ratchathani, where multiple transitions were observed, aligning with studies from Thailand and broader regional trends documented in the literature (Newby et al., 2014; Burra et al., 2021; Promkhambut et al., 2023; Zafar et al., 2025). As agricultural development, damming, and mining alter habitats, species like the secondary dengue vector *Ae. albopictus* flourish, raising disease transmission risks (Vallée et al., 2009; Jeefoo et al., 2011; Tangena et al., 2017a; Weterings et al., 2018; Lim et al., 2020; Soukavong et al., 2024; Zafar et al., 2025).

Urbanization across all provinces proceeded at a relatively slow rate (0.4–3.8% over the study period), with growth concentrated around major urban centers. The spread of artificial breeding sites, including water storage containers, discarded tires, and clogged drains, and the rise in human population density promote *Aedes* spp. growth and rapid transmission of dengue viruses. Urban challenges such as the urban heat island effect, flash floods, habitat loss, and water pollution could be reduced through landscape management strategies, such as restoring and maintaining urban green spaces. These areas provide shade and evapotranspiration, helping to moderate or control temperature extremes and deliver ecosystem services like rainfall interception and infiltration, which help lower urban flood risks (Highfield and Brody, 2013). Furthermore, green spaces can also help decrease dengue transmission.

The development of large, permanent, or semi-permanent water bodies, the alteration of water flow regimes, and the potential increase in *Aedes* spp. breeding sites along lake margins or within associated irrigation systems contribute to the situation. The construction of hydropower dams in Champasak and Savannakhet provinces in Laos has increased wetland areas; while ecologically valuable, these can also create new or enlarged breeding habitats, particularly for certain species of *Aedes*, if not properly managed (Sarfraz et al., 2012). This dual role underscores the complexity of wetland management, necessitating a balance between ecological benefits and public health concerns.

Our findings clearly indicate how LULC change impacts ecosystems and habitat distribution. Replacing natural forests with rubber plantations and cropland decreases biodiversity and habitat variety, while also changing microclimatic conditions that support *Ae. albopictus* (secondary dengue vector) populations and their predators. Expanding built-up areas further fragments landscapes and reduces ecological connectivity, leading to habitat loss (Liu et al., 2016). Conversely, the growth of rice fields and wetlands creates new ecological niches that can benefit some species (Natuhara, 2013), such as disease vectors including dengue and malaria (Yasuoka and Levins, 2007; Jarju et al., 2009; Conlan et al., 2015) but displace others, e.g. amphibians and certain bird species that depend on undisturbed terrestrial or forest habitats (Conlan et al., 2015). These results demonstrate that land cover changes are not merely surface modifications but key drivers of ecological restructuring that influence habitat distribution and resilience (Prokopová et al., 2019). In summary, our findings suggest that LULC changes, such as deforestation, agricultural expansion, and urban development, alter ecological conditions that may impact dengue risk. Although this study does not establish a direct causal link, the observed trends align with broader literature emphasizing the potential for such modifications to increase dengue risk (Thammapalo, 2005; Walsh et al., 2006; Tangena et al., 2017a; Rahman et al., 2021; Zafar et al., 2021, 2025). The main limitation of this analysis is that it serves as a complementary contribution rather than a definitive causal assessment. LULC changes affect different areas in diverse ways, and understanding their relationship with dengue risk requires coordinated efforts among environmental, land management, and public health agencies. Additionally, our analysis does not include key factors of dengue transmission, such as vector abundance, breeding-site characteristics, human mobility, and laboratory-confirmed epidemiological data, which may influence the observed patterns. Other important drivers, such as climate variability, population dynamics, and healthcare access are also important and have been examined in previous studies (Wongkoon et al., 2011; CDCD, 2019; Doum et al., 2020; Senavong et al., 2021; Zafar et al., 2021, 2025). Future research should combine LULC analysis with entomological surveys, high-resolution epidemiological data, and more detailed LULC datasets to improve causal understanding and clarify the mechanisms linking landscape change with dengue risk. Overall, our findings should be viewed as complementary, highlighting the importance of collaboration among health, environmental, and land management agencies in both countries.

## 5 Conclusions

This study adds to the growing evidence on the ecological impacts of LULC changes and their potential to influence vector-borne disease dynamics, especially dengue. The expansion of rubber plantations, forest clearing, and urban growth are key factors driving ecological transformation in Laos and Thailand. Combining spatial analysis of LULC changes with epidemiological data will be essential in future research to better understand how environmental factors and disease spread are connected. Future conservation efforts should also focus on preserving ecosystem integrity to lower the risk of emerging diseases. Implementing tailored land management policies, sustainable urban planning, and targeted vector control strategies will be vital to reducing the potential health risks linked to these changes.

## Ethical approval

Not applicable.

## CRediT authorship contribution statement

**Muhammad Umar:** Conceptualization, Methodology, Formal analysis, Investigation, Resources, Data curation, Writing – original draft, Writing – review & editing. **Sobia Asghar:** Investigation, Resources, Data curation, Writing – review & editing. **Sumaira Zafar:** Conceptualization, Methodology, Resources, Supervision, Validation, Visualization, Writing – review & editing.

## Statement on the use of AI-assisted technologies

During the preparation of this work, the authors used Grammarly and Gemini in order to correct the grammar and sentence structure. After using these tools, the authors reviewed and edited the content as needed and take full responsibility for the content of the published article.

## Funding

This research did not receive any specific grant from funding agencies in the public, commercial, or not-for-profit sectors.

## Declaration of competing interests

The authors declare that they have no known competing financial interests or personal relationships that could have appeared to influence the work reported in this paper.

## Data Availability

The data supporting the conclusions of this article are included within the article and its supplementary files. Raw data are available upon request.

## Appendix A Supplementary data

Supplementary file 1 Overall classification accuracy was measured with confusion matrices for all the provinces and years. Supplementary file 1

Supplementary file 2 Land use and land cover (LULC) changes in % from 2001 to 2025 in Mukdahan and Ubon Ratchathani (Thailand) and Champasak and Savannakhet (Laos). Supplementary file 2
